# London Lock Hospital

**Published:** 1902-08-02

**Authors:** 


### Around the Hospitals.

### LONDON LOCK HOSPITAL.

### THE YEAR'S WORK:

#### The Female Hospital.

The Female Hospital in Harrow Road has not been so full for many years as it was in 1901, when the average number of beds occupied throughout the year was 98. The additional accommodation of 14 beds provided by the reopening of the Prince of Wales's Ward was fully taken advantage of. The nursing staff has also been added to. Every case has been admitted without letter, and applicants of every class, age, and state of destitution have been received. The majority of the patients come from the Metropolitan area, but many have been only a short time in London. New subscribers are greatly needed to enable the governors to take in all who apply. The total number of inmates of the Rescue Home attached to the Female Hospital was 111, including 61 who were in the home at the beginning of the year. Of these, 30 were sent to service, 16 were restored to friends, 9 were sent to other homes, and 11 were unsuitable cases. Eleven of the girls placed in service have received one guinea each for remaining from one to two years, and in all these cases mistresses have testified as to their good conduct. The laundry has earned £810, an increase of £77 on the previous year. "The idea," says the report, "of having a rescue home adjacent to the hospital, and of taking in there all the suitable cases when relieved, has been, and is, a special characteristic of our work, and makes ours the only institution of its kind in the Kingdom. It has been an unspeakable gain, whether from a physical, moral or spiritual point of view."

The total ordinary receipts of the female hospital were £5,606. This includes subscriptions, £783; donations, £1,160; Sunday fund, £208 6s. 8d.; Saturday fund, £25; contributions by parishes on behalf of patients, £2,636 15s. 3d. Mrs. Bramwell Booth has become an annual subscriber. The total ordinary expenditure was £4,741 17s. 2d. Repairs to chimney-stacks cost £269, and there was a balance of income over expenditure of £2,543 12s. 7d. The rescue home had a deficit of £364 14s. 7d.

#### The Male Hospital and Out-Patients' Departments.

The number of male patients admitted was 257. Of out-patients, 14,997 old and 4,779 new males were seen, and 2,060 old and 414 new females. The attendances of out-patients were 2,259 more than in 1900. There was a decrease of in-patients in December. The City Missionary alluded to above visits the wards and waiting-rooms almost daily.

The ordinary income was £2,914, and there were no legacies in this department. The total expenditure was £2,377 and a balance of £536 appears on the right side. The debt on the institution, which was £3,582, was reduced in 1891 to £866, owing to the help received by means of legacies.

The Bishop of London visited the hospital and home in October, and was much pleased with all he saw.

